# Laparoscopic two-stage operation for rectal cancer with refractory obstructive colitis after kidney transplantation: a case report

**DOI:** 10.1186/s40792-020-0798-z

**Published:** 2020-02-03

**Authors:** Atsuro Fujinaga, Tomonori Akagi, Tsuyoshi Etoh, Kazuhiro Tada, Yusuke Itai, Yohei Kono, Takahiro Hiratsuka, Kosuke Suzuki, Tomotaka Shibata, Yoshitake Ueda, Manabu Toujigamori, Hidefumi Shiroshita, Norio Shiraishi, Masafumi Inomata

**Affiliations:** 1 Department of Gastroenterological and Pediatric Surgery, Oita University Faculty of Medicine, Oita, Japan; 2 Department of Comprehensive Surgery for Community Medicine, Oita University Faculty of Medicine, Oita, Japan

**Keywords:** Colorectal cancer, Kidney transplantation, Obstructive colitis

## Abstract

**Background:**

Although obstructive colitis with colon cancer is not a rare disease, most cases can be improved with conservative therapy. We report a case of a patient who underwent a laparoscopic two-stage operation for rectal cancer with refractory obstructive colitis after kidney transplantation.

**Case presentation:**

The patient was a 71-year-old man taking immunosuppressants who had previously undergone right living kidney transplantation for chronic nephritis. He presented to hospital complaining of abdominal pain and was diagnosed as having rectal cancer with obstructive colitis. Although conservative therapy by fasting was continued for 5 weeks, his obstructive colitis did not improve. Therefore, we decided to perform a two-stage operation. First, we performed a laparoscopic Hartmann’s operation. It took 6 months for his obstructive colitis to improve after this operation, and then we performed a laparoscopic colorectal anastomosis. There were no postoperative complications in either operation.

**Conclusion:**

A laparoscopic two-stage operation could be one of the operative options to reduce postoperative complications in patients with comorbidities such as taking immunosuppressants.

## Background

Obstructive colitis is an ulcero-inflammatory and necrotizing condition that occurs in the colon proximal to a stenotic lesion such as colon cancer [1]. It usually improves within 5 weeks with conservative therapy [2, 3], but it can be refractory to treatment with the long-term use of immunosuppressants. Here, we report a case of a patient with refractory obstructive colitis after kidney transplantation who underwent a laparoscopic two-stage operation for treatment of rectal cancer.

## Case presentation

A 71-year-old Japanese man had undergone laparoscopic right living-donor kidney transplantation for end-stage renal disease due to chronic nephritis at the age of 65 years. His immunosuppression regimen included combination therapy with tacrolimus 1 mg/day, mycophenolate mofetil 1000 mg/day, and prednisolone 4 mg/day. Renal graft function remained stable without any episodes of rejection.

**Fig. 1:**
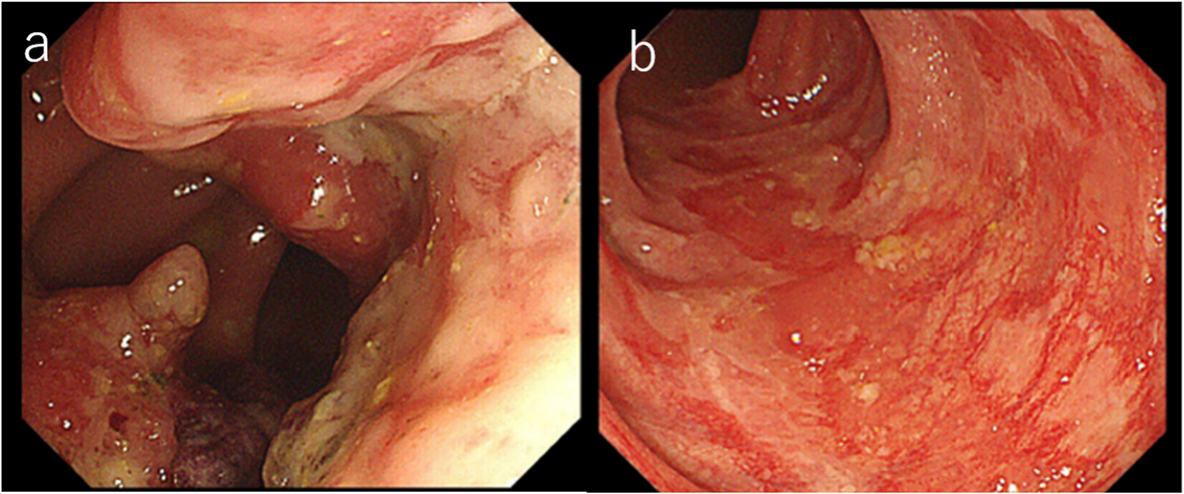
Colonoscopy revealed a near-circumferential mass at the rectosigmoid colon (**a**). The descending colon on the oral side of the tumor showed reddening and edematous changes circumferentially (**b**)

Six years after transplantation, he presented to our hospital complaining of abdominal pain. A colonoscopy revealed a near-circumferential mass at the rectosigmoid colon. The tumor proved to be adenocarcinoma (tub) by biopsy. The descending colon on the oral side of the tumor showed reddening and edematous changes circumferentially (Fig. 1a, b). The colitic region was observed through the normal mucous membrane from 8 to 30 cm on the oral side of the tumor. The most oral side of the reddening and edematous change was located at sigmoid-descending colon junction. Histological examination by biopsy revealed that the colonic mucosa of the colitic region showed only moderate colitis. There was no evidence of cytomegalovirus colitis. He had a previous history of hypertension but no cigarette smoking or drinking. His family history showed no remarkable findings.

**Fig. 2:**
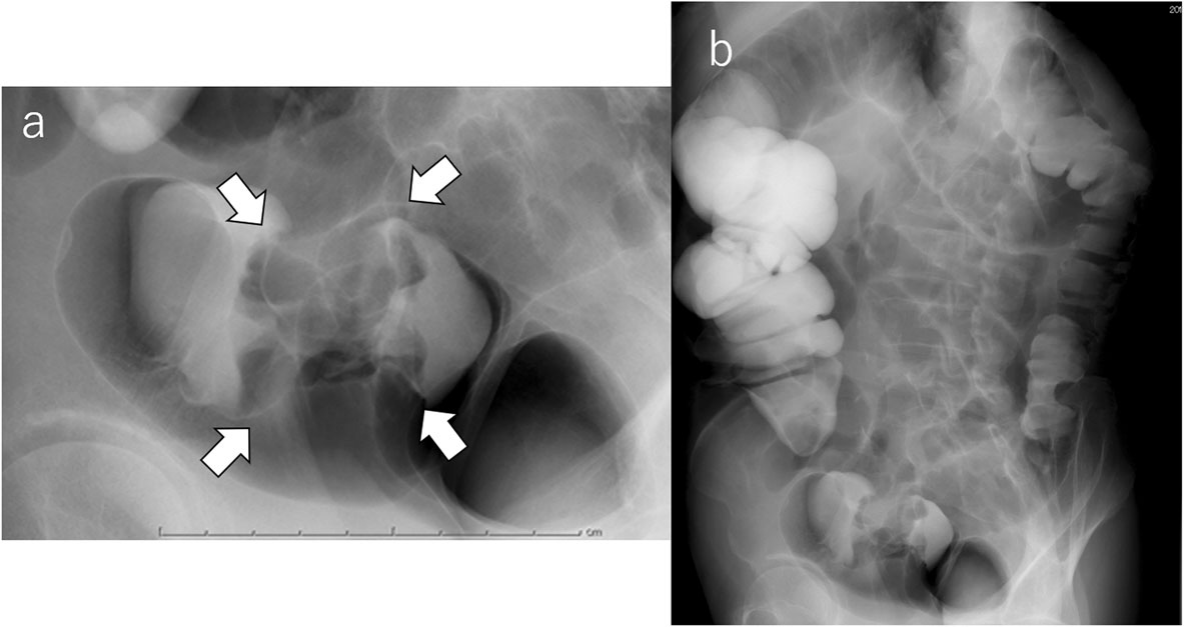
Barium enema examination showed a tumor measuring 45 mm in diameter at the rectosigmoid colon (**a**, arrow). No other colorectal lesions were observed (**b**)

**Fig. 3:**
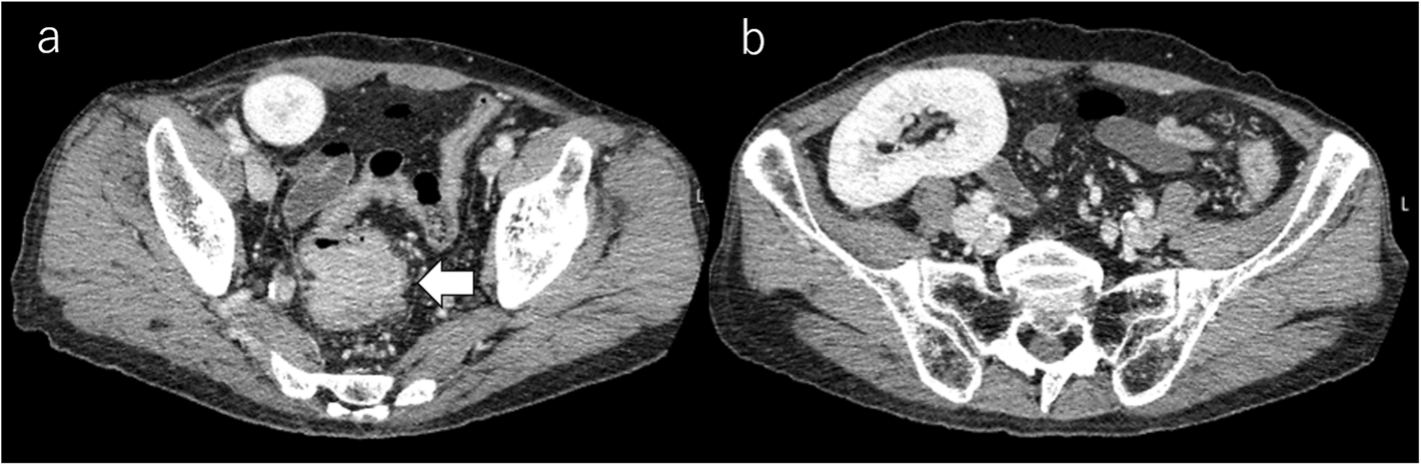
A CT scan revealed a circumferential mass with irregular surface and contrast effect and wall thickening at the rectosigmoid colon (**a**, arrow) and the transplanted kidney located in the right pelvic cavity (**b**)

On admission, his abdomen was flat and soft, and abdominal pain disappeared during fasting. No special signs were noted except for a healed surgery scar on his right abdominal wall. Laboratory tests showed no anemia, serum creatinine was 1.1 μmol/L, liver function was normal, and serum tumor markers (CEA, CA19-9) were within normal range. A barium enema examination showed a tumor measuring 45 mm in diameter at the rectosigmoid colon. No other colorectal lesions were observed (Fig. 2a, b). A subsequent computed tomography (CT) scan revealed a circumferential mass with irregular surface and contrast effect and wall thickening at the rectosigmoid colon. The transplanted kidney was located in the right pelvic cavity (Fig. 3a, b). No evidence of lymph node or distant metastasis was apparent. Conservative therapy of fasting and laxatives without a decompression tube improved his symptoms. Patient continued to take immunosuppressants during the fasting period. After 5 weeks of conservative therapy, a colonoscopy revealed the same findings as the previous test. Considering the long-term use of immunosuppressants and the persistence of the obstructive colitis, we decided to perform a two-stage operation and performed a laparoscopic Hartmann’s operation first. The patient took the immunosuppressant on the day of surgery and resumed taking it from the first day after the operation.

**Fig. 4:**
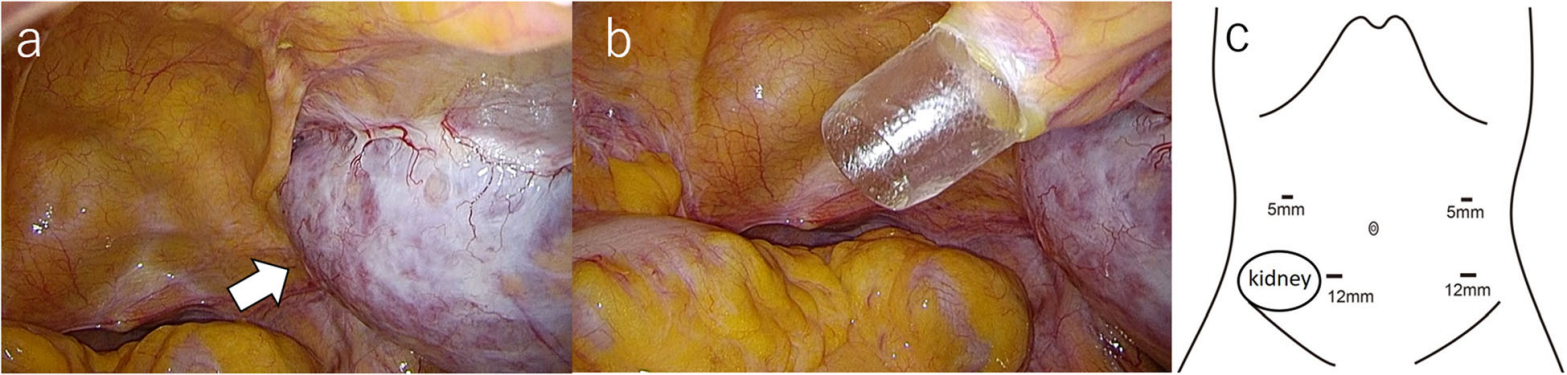
The transplanted kidney was located in the right lower abdomen (**a**, arrow). The right lower quadrant port was inserted more towards the midline than usual to avoid the transplanted kidney (**b**, **c**)

**Fig. 5:**
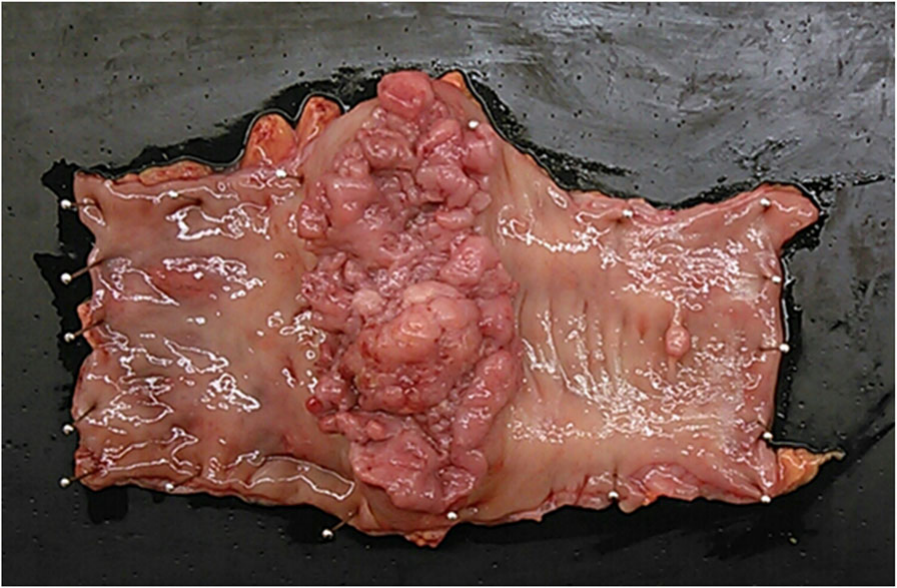
The specimen was a circumferential tumor measuring 90 × 40 mm. The mucosa except for the tumor of the specimen had no evidence of colitis

After induction of general anesthesia, the patient was positioned in the lithotomy position. Complete exploration of the abdominal cavity suggested that there was no liver or peritoneal carcinomatosis. The tumor was identified at the rectosigmoid colon location, and the transplanted kidney was also located in the right lower abdomen (Fig. 4a). To avoid the transplanted kidney, we inserted the right lower quadrant port more towards the midline than usual (Fig. 4b, c). Considering the effect of abdominal pressure on the transplanted kidney, the pneumoperitoneum pressure was set at 10 mmHg. The resections performed achieved complete tumor removal with tumor-free margins and tumor-specific mesorectal excision. We cut the superior rectal artery, preserved the left colic artery, and harvested the D3 lymph nodes. The distal rectum was transected intracorporeally and then a 6-cm left supraumbilical incision was made to remove the proximal colon. We dissected the proximal sigmoid colon and removed the tumor. Then, we confirmed the difference between normal mucosa and colitic region. Finally, a sigmoid colostomy was performed. The operation was completed successfully with an operating time of 206 min and blood loss of 60 mL. The specimen was a circumferential tumor measuring 90 × 40 mm. The mucosa except for the tumor in the specimen was normal (Fig. 5). Histopathology revealed a well-to-moderately differentiated adenocarcinoma with invasion through the muscularis propria into pericolorectal tissues. The resection margins were free, and there were no lymph node metastases, indicating a tumor stage of pT3N0M0.

**Fig. 6:**
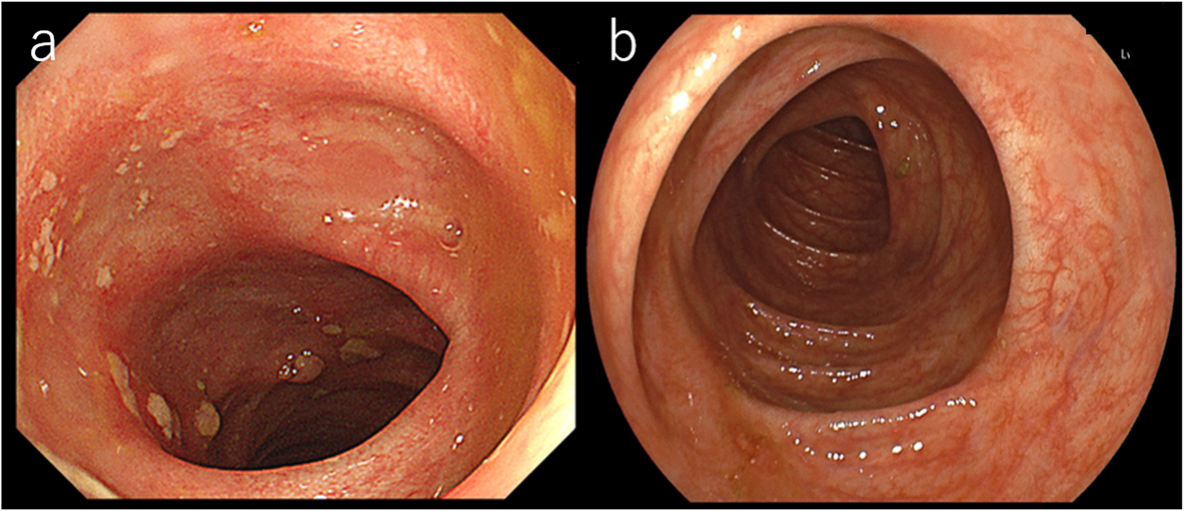
**a** Colonoscopy on 3 months after surgery. The reddening and edematous changes are still remained on the descending colon. **b** Colonoscopy on 6 months after surgery. Obstructive colitis improved

Postoperative course was uneventful. The patient needed time to learn the stoma application and discharged 30 days after surgery. At 3 months after the surgery, obstructive colitis still remained. At 6 months after the surgery, his obstructive colitis had improved (Fig. 6a, b), and we performed a laparoscopic colorectal anastomosis as the second stage.

After induction of general anesthesia, the colostomy was returned to the abdominal cavity. GelPort® (Applied Medical, Rancho Santa Margarita, CA) with three ports (12 mm, 5 mm, 5 mm) was attached where the colostomy was, and operation was started laparoscopically. There was a little adhesion in the abdominal cavity and the stump of the rectum was easily found. An anastomosis was performed using an autosuture device. The operating time was 80 min and blood loss was 5 mL.

The patient discharged hospital 4 days after second surgery with no postoperative complications. During 1 year of follow-up, no allograft rejection, complications, or recurrence was observed.

## Discussion

To our knowledge, there are no reports of obstructive colitis due to rectal cancer after kidney transplantation. In the present case, fasting as decompression therapy was carried out for 5 weeks before the first operation, but the obstructive colitis still remained. Considering this patient’s long-term use of immunosuppressants, we decided on less invasive treatment, a laparoscopic two-stage operation to reduce postoperative complications. Then, we could successfully treat the patient without any intra- or postoperative complications for the patient with comorbidities.

The incidence of obstructive colitis is about 1–2 % among all colorectal cancers [4, 5]. Symptoms include abdominal pain, bloody stool, and diarrhea, and symptoms associated with intestinal ischemia are characteristic of this condition [6]. The cause of obstructive colitis is the ischemia due to impairment of blood supply secondary to elevation of the endoluminal pressure, distension of the colonic wall, and other factors which impair adequate perfusion [6]. Therefore, histological findings suggest that obstructive colitis is similar to ischemic colitis, and it usually improves within 5 weeks of conservative therapy if effective conservative therapy is performed [2, 3], but the success rate of this therapy for obstructive colitis is unclear, and the number of cases for which conservative therapy is ineffective is unknown. On the other hand, once patients taking immunosuppressants have enteritis, enteritis have been prolonged [7, 8]. In this patient, colitis was prolonged and the speculated cause of this may be keeping immunosuppressants. Among the several conservative therapies including fasting, colorectal stenting, and a decompression tube, it is unclear which therapy is best from the viewpoints of safety, efficacy, and oncology [9, 10]. In our patient, since his symptom disappeared with fasting and his bowel movements were good, we judged fasting to be effective.

Long-term use of immunosuppressants, which are essential after transplantation, is one of the risk factors of suture failure or delay of wound healing, and the incidence of suture failure is reported to be 6.8% in patients taking immunosuppressants compared with 3.3% in patients not taking them [11]. Further, the suture failure rate when anastomosis is performed in an ulcerated area is reported to be 25% [12]. Wound healing was reported to be poor at the anastomosis in a rat model administered immunosuppressants [13]. To prevent complications including suture failure, we planned to perform two-stage operation and confirm the area of colitic region during operation.

Surgery in kidney transplant patients can be difficult due to narrowness in the abdominal cavity caused by the transplanted kidney. However, a laparoscopic operation is technically feasible by shifting the position of the port [14]. Laparoscopic surgery was performed in four patients with colorectal cancer after kidney transplantation. Operative times and blood loss were not inferior to those of open surgery and the length of the hospital stays was significantly shorter with laparoscopic surgery. Further, no postoperative complications or loss of the transplanted kidney occurred [15]. In addition, laparoscopic surgery for advanced rectal cancer is better in terms of blood loss, length of hospital stay, and wound infection than open surgery, and thus is considered useful for high-risk cases [16, 17].

As the taking of immunosuppressants progresses, death with functioning graft, which causes death due to cardiovascular disease, malignancy, or other conditions while the transplanted kidney remains functional, can be a problem rather than loss of the transplanted kidney due to rejection [18]. The risk of developing colorectal cancer during immunosuppressant therapy is reported to be 2 to 5 times higher than that when immunosuppressants are not taken [19]. Recently, the number of colorectal cancer operations has been increasing in Japan [20], and in the future, the number of patients with colorectal cancer who are taking immunosuppressants is expected to increase. Clinicians should keep in mind that conservative therapy might be ineffective, and thus the appropriate choice of a therapeutic strategy with less invasive surgery is important in such patients. From the above, the laparoscopic two-stage operation performed in this case appeared to be useful.

## Conclusions

A laparoscopic two-stage operation could be one of the operative options to reduce postoperative complications in patients with comorbidities such as taking immunosuppressants.

## Data Availability

The data are not available for public access because of patient privacy concerns but are available from the corresponding author on reasonable request.
